# Structural basis for reversible amyloids of hnRNPA1 elucidates their role in stress granule assembly

**DOI:** 10.1038/s41467-019-09902-7

**Published:** 2019-05-01

**Authors:** Xinrui Gui, Feng Luo, Yichen Li, Heng Zhou, Zhenheng Qin, Zhenying Liu, Jinge Gu, Muyun Xie, Kun Zhao, Bin Dai, Woo Shik Shin, Jianhua He, Lin He, Lin Jiang, Minglei Zhao, Bo Sun, Xueming Li, Cong Liu, Dan Li

**Affiliations:** 1 Interdisciplinary Research Center on Biology and Chemistry, Shanghai Institute of Organic Chemistry, Chinese Academy of Sciences, Shanghai, 201210 China; 2 University of Chinese Academy of Sciences, Beijing, 100049 China; 3 Bio-X Institutes, Key Laboratory for the Genetics of Developmental and Neuropsychiatric Disorders, Ministry of Education, Shanghai Jiao Tong University, Shanghai, China; 4 Beijing Advanced Innovation Center for Structural Biology, Tsinghua-Peking Joint Center for Life Sciences, School of Life Sciences, Tsinghua University, Beijing, China; 5 School of Life Science and Technology, ShanghaiTech University, Shanghai, 201210 China; 6 Shanghai Institute of Applied Physics, Chinese Academy of Sciences, 239 Zhang Heng Road, Pudong New District, Shanghai, 201203 China; 7 Department of Neurology, Molecular Biology Institute, and Brain Research Institute, UCLA, Los Angeles, CA 90095 USA; 8 Department of Biochemistry and Molecular Biology, the University of Chicago, Chicago, IL 60637 USA

**Keywords:** RNA-binding proteins, Stress granules, Protein aggregation, Electron microscopy, X-ray crystallography

## Abstract

Subcellular membrane-less organelles consist of proteins with low complexity domains. Many of them, such as hnRNPA1, can assemble into both a polydisperse liquid phase and an ordered solid phase of amyloid fibril. The former mirrors biological granule assembly, while the latter is usually associated with neurodegenerative disease. Here, we observe a reversible amyloid formation of hnRNPA1 that synchronizes with liquid–liquid phase separation, regulates the fluidity and mobility of the liquid-like droplets, and facilitates the recruitment of hnRNPA1 into stress granules. We identify the reversible amyloid-forming cores of hnRNPA1 (named hnRACs). The atomic structures of hnRACs reveal a distinct feature of stacking Asp residues, which contributes to fibril reversibility and explains the irreversible pathological fibril formation caused by the Asp mutations identified in familial ALS. Our work characterizes the structural diversity and heterogeneity of reversible amyloid fibrils and illuminates the biological function of reversible amyloid formation in protein phase separation.

## Abstract (web summary)

Low complexity (LC) domains can drive the formation of both amyloid fibrils and protein droplets. Here, the authors identify reversible amyloid cores from the LC of hnRNPA1, based on which they elucidate the structural basis of reversible fibrillation and its interplay with hnRNPA1 droplet formation.

## Introduction

Membrane-less organelles are organizations of proteins, RNA, or DNA molecules that condense at specific subcellular loci to fulfill defined biological functions^1,2^. In contrast to steady membrane-bound organelles, many membrane-less organelles, such as stress granules and P-bodies, dynamically assemble and disassemble in response to cellular stress and other stimuli^3,4^. Extensive studies have identified that many RNA-binding proteins, such as FUS, hnRNPA1, and TDP43, largely contribute to this conditional cellular compartmentation^5,6^. These proteins commonly contain low complexity (LC) domains that can drive the full-length proteins to undergo liquid–liquid phase separation (LLPS) via weak multivalent interactions^7–9^. On the other hand, many LCs are also prone to amyloid aggregation^10,11^. Missense mutations of LCs identified in neurodegenerative diseases, including amyotrophic lateral sclerosis (ALS), multisystem proteinopathy (MSP), and frontotemporal dementia (FTD), can promote amyloid aggregation, which is closely associated with the pathogenesis of these diseases^12–14^.

Liquid-like droplets and amyloid fibrils are both higher-ordered protein assemblies, while are different in thermodynamics and in principle can form successively. Indeed, confocal microscopy has shown amyloid fibrils grow out from liquid-like droplets of hnRNPA1 and FUS^15,16^. In the sense that amyloid fibrils are highly stable and resistant to proteolysis and harsh conditions (e.g., SDS and heating), amyloid-prone property seems to be of a high risk for cells and might not be the basis for dynamic granule assembly^17–19^. However, it has been recently emerging that amyloid fibrils formed by LC may be metastable and even readily reversible. Mcknight et al. showed that RNA-binding proteins including FUS and hnRNPA1 can form SDS-sensitive metastable fibrils^20–22^. Eisenberg et al. observed temperature-sensitive fibrils formed by an artificial sequence derived from LC^23^. Our previous work on FUS LC identified two segments that form reversible fibrils regulated by temperature and phosphorylation^24^. These observations altered the view of amyloid fibrils as thermostable end-product of protein super-molecular assembly, and raised the possibility that amyloid formation may play a functional role in the assembly of membrane-less organelles.

In this work, we directly visualize temperature-sensitive amyloid fibrils in the liquid-like droplets of hnRNPA1 by transmission electron microscopy (TEM). The fibrils synchronize with the occurrence and disappearance of liquid-like droplets as we observe, while over time, irreversible fibrils form and accumulate. We identify a reversible amyloid core from hnRNPA1 LC (named hnRAC1) and monitor the real-time in situ self-disassembly of hnRAC1 fibrils by atomic force microscopy (AFM). We further determine the atomic structure of hnRAC1 by microelectron and X-ray diffraction to the resolution of 1.0 Å and 1.4 Å, respectively. The structure reveals a distinct feature of negatively charged Asp residues stacking along the fibril, which contributes to fibril instability. Based on this structure, we identify another two reversible amyloid cores from hnRNPA1 LC (named hnRAC2 and hnRAC3) and more from hnRNP families. Asp mutations found in familial ALS enhance the formation of irreversible fibrils. We show that hnRACs not only define the formation of reversible fibrils but also reinforce hnRNPA1 phase separation in vitro and involvement into stress granules in vivo. Our work bridges the liquid phase and solid phase of hnRNPA1 and illuminates the functional role of reversible amyloids in the assembly of stress granules and the pathological risk as intermediates en route to irreversible fibrils.

## Results

### Multiple-state self-assembly of hnRNPA1

**Fig. 1:**
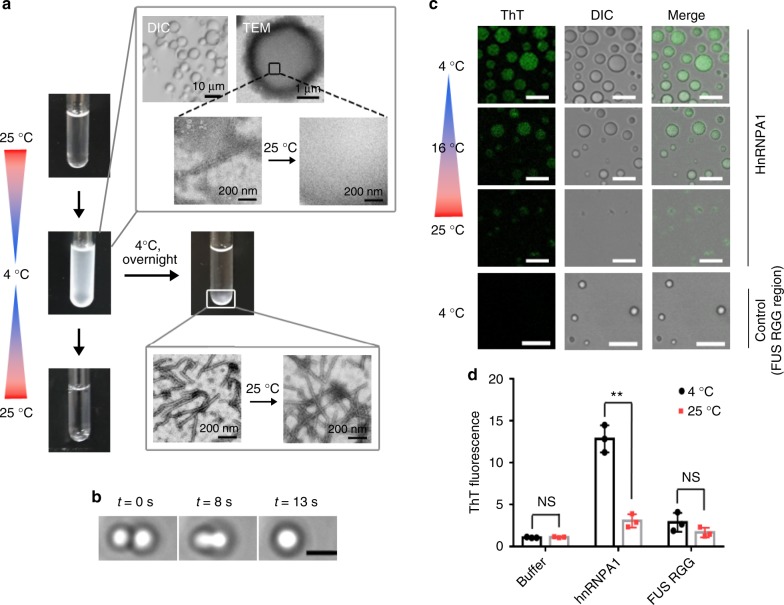
Phase separation and amyloid fibril formation of hnRNPA1. **a** Phase transition of hnRNPA1 upon temperature and time. Liquid-like droplets formed at 4 °C were imaged by DIC microscopy. Amyloid fibrils were imaged by negative-staining TEM. **b** Representative montage of hnRNPA1 droplet fusion detected by optical tweezers. The scale bar is 2.5 μm. **c** In-situ imaging of hnRNPA1 phase separation as temperature increased by DIC microscopy and fluorescence microscopy. Control images are in the same gray color depth as the hnRNPA1 images. Scale bars are 10 μm. **d** ThT fluorescence of hnRNPA1 and control (FUS RGG) samples at 4 °C and 25 °C was measured by fluorescence spectrophotometry. Data shown are means ± s.d., with *n* = 3 independent samples. Values were compared using Student’s *t* test. **p < 0.01. NS represents non-significant. Source data are provided as a Source Data file

To observe the phase separation of hnRNPA1, we cooled down the hnRNPA1 solution from 25 °C to 4 °C. The solution turned cloudy in a few minutes. Differential interference contrast (DIC) microscopy showed spherical droplets of hnRNPA1 in the liquid phase (Fig. 1a). The liquid property of the droplets was shown as that two adjacent droplets fused spontaneously within seconds measured by optical tweezers (Fig. 1b). We deposited the phase-separated hnRNPA1 sample on TEM grids to check the content of the droplets by negative-staining TEM. Intriguingly, we observed bunches of amyloid fibrils in the droplets (Fig. 1a; Supplementary Fig. 1). Although we carefully monitored the size and shape of droplets during the TEM grid preparation to minimize the influence of drying, it is still possible that the observed fibrils are derived from a drying artifact. Thus, we further validated the amyloid fibril formation by monitoring Thioflavin T (ThT) fluorescence using confocal microscopy and spectrophotometry (Fig. 1c, d). The results showed a specific enhancement of ThT intensity in the droplets, which consistent with the TEM imaging, indicates amyloid fibril formation in the droplets. To rule out a possible artifact of ThT staining, the FUS RGG region (residues 371–526) was used as a control. FUS RGG can undergo LLPS, but contains no amyloid-forming sequence. The control sample showed no obvious ThT intensity comparing with the hnRNPA1 sample (Fig. 1c), and artificial enhancement of ThT intensity by adjusting the image contrast showed that ThT spread out in the control sample, rather than condensed in the droplets as that seen in the hnRNPA1 sample (Fig. 1c; Supplementary Fig. 2).

As temperature reverted back to 25 °C, the hnRNPA1 solution became clear again, and neither droplet nor amyloid fibril was observed, indicating that the fibrils are reversible (Fig. 1a, c). As we estimated based on the ThT intensity, <10% of the total proteins in molecules formed reversible fibrils (Supplementary Fig. 3). Note that ThT fluorescence revealed a small amount of amyloid aggregates remained as temperature increased back to 25 °C (Fig. 1c), which indicates that a portion of fibrils maturated to relatively irreversible fibrils. Moreover, as we kept the cloudy hnRNPA1 solution at 4 °C for hours, the solution spontaneously became clear and the amount of reversible fibrils decreased accordingly (Fig. 1a; and Supplementary Fig. 3). Overnight, a significant amount of irreversible amyloid fibrils emerged which are stable as temperature increased to 25 °C for elongated time (Fig. 1a).

In these experiments, we observed three states of hnRNPA1 assemblies: liquid-like droplets, reversible fibrils, and irreversible fibrils. Among them, irreversible fibrils are known to be constructed by folded and highly ordered stacking of proteins, such that they are the most thermodynamic and hard to dissemble. Liquid-like droplets and reversible fibrils appear to be dynamic assemblies that can self-dissociate into soluble proteins or over time, proceed to enter the thermostable state of irreversible fibrils depending on energy input (e.g., temperature).

### Identification of a reversible amyloid core of hnRNPA1

**Fig. 2:**
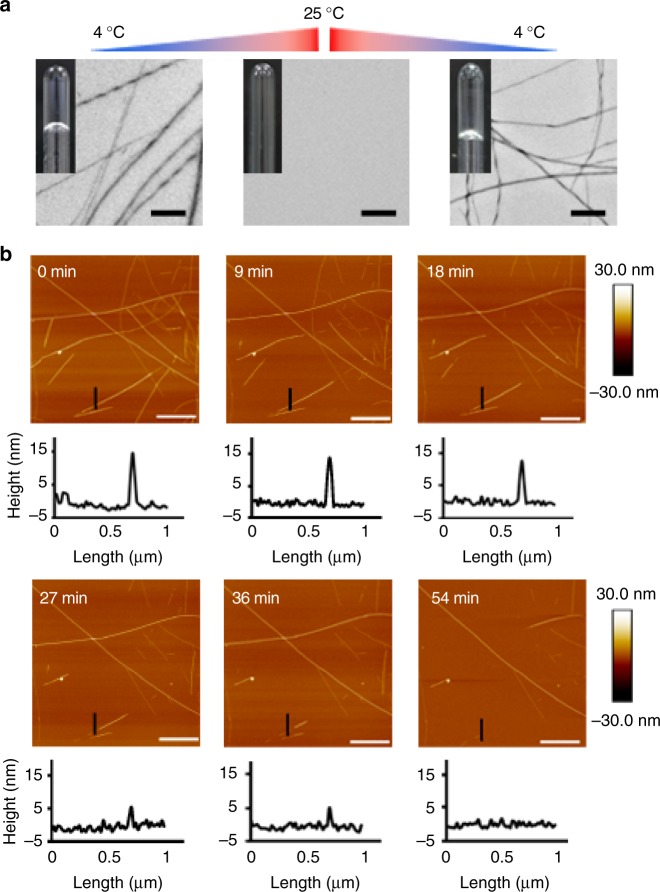
Reversible amyloid fibrils formed by hnRNPA1 RAC1. **a** Reversible amyloid fibrils and hydrogels formed by hnRAC1 regulated by temperature. Amyloid fibrils were imaged by TEM. Scale bars are 500 nm. **b** Montage of real-time and in situ visualization of the self-dissociation of hnRAC1 fibrils by AFM. The black short line on AFM images indicates the cross-section that is analyzed below. The bright spot on AFM images indicates the imaging position. Scale bars are 2.0 μm

We next sought to investigate the mechanism underlying the reversible amyloid formation of hnRNPA1. To identify the reversible amyloid core (RAC) of hnRNPA1, we synthesized a series of segments selected from the LC domain with different lengths from 5 to 12 residues. The segments contain [S/G]Y/F[S/G] and/or RGG motifs that are characteristic in LC^21,25–27^ (Supplementary Fig. 4). The segments were screened for reversible amyloid formation. The result showed that segment ^209^GFGGNDNFG^217^ (named hnRAC1), but not the others, formed hydrogel at 4 °C. The hydrogel was composed of amyloid fibrils observed by TEM (Fig. 2a). As temperature increased to 25 °C, the fibrils disassociated spontaneously, resembling the behavior of full-length hnRNPA1 (Figs. 1a, 2a). Moreover, we utilized AFM to monitor the real-time and in situ self-disassembly of hnRAC1 fibrils at a single-fibril level. We observed that hnRAC1 fibrils gradually melt down in a timescale of minutes upon temperature increased from 4 °C to room temperature, whereas irreversible fibrils stayed unchanged (Fig. 2b; Supplementary Fig. 5). This observation emphasizes the remarkably dynamic property of reversible fibrils that is distinct from irreversible fibrils extensively studied previously^18,19,28,29^, although X-ray fibril diffraction showed that reversible hnRAC1 fibrils also feature a typical cross-β architecture as seen in irreversible amyloid fibrils^19,28^, with an interstrand spacing of ~4.8 Å and an inter-sheet spacing of ~11 Å (Supplementary Fig. 6).

### Atomic structures of hnRAC1

**Fig. 3:**
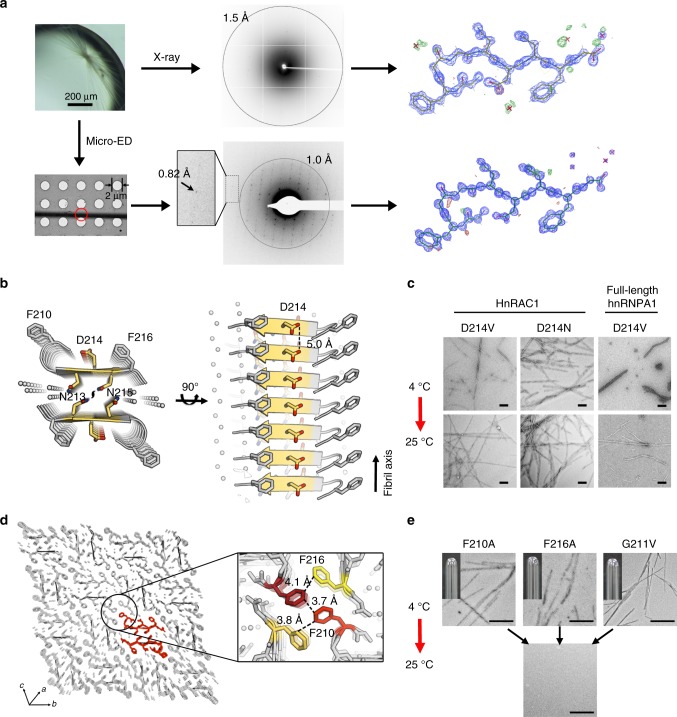
Structure determination and mutagenesis of hnRAC1. **a** Atomic structures of hnRAC1 determined by micro-ED and X-ray diffraction. The red circle on EM grid indicates the shooting spot of electron beam. HnRAC1 crystals diffracted electron beam to the highest resolution of 0.82 Å. The structure of hnRAC1 was solved at the resolution of 1.0 Å by micro-ED and 1.4 Å by X-ray diffraction. Structure models and density maps of hnRAC1 were shown. 2*Fo*-*Fc* maps are contoured at 2.0 rmsd (blue). *Fo*-*Fc* maps are contoured at 3.0 rmsd (green and red). Red crosses represent water. **b** The atomic structure of hnRAC1 in the fibrillar form. The structure features a cross-β architecture with a hydrophilic interface composed of N213 and N215, that together with D214, form the fibril core (colored in yellow). Residue side chains are shown as sticks. Nitrogen atoms are in blue. Oxygen atoms are in red. Water molecules are shown as spheres. The two-fold screw axis is indicated. The distance between Asp residues from neighboring β-strands in the same sheet is indicated. **c** Effects of D214 mutations on hnRAC1 and full-length hnRNPA1 fibril reversibility. The mutations resulted in fibrils that unlike the reversible wild-type fibrils remained stable as warmed up to 25 °C. Scale bars are 200 nm. **d** The crystal lattice of hnRAC1 viewed down the fibril sheets. The unit of fibril spine is colored in red. Inter-fibrillar π–π interactions are shown in the zoom-in view. Phe residues involved in π–π interactions are from four neighboring hnRAC1 molecules. Distances between Phe residues are indicated. F210 residues are colored in red. F216 residues are in yellow. **e** Effects of inter-fibrillar interactions on hydrogel formation. Mutations of F210A, F216A, and G211V all disrupted the formation of hydrogels, whereas the mutants can still form reversible fibrils. Scale bars are 200 nm

To reveal the structural basis underlying the reversible amyloid formation of hnRAC1, we determined the atomic structure of hnRAC1 in the fibrillar form to the resolution of 1.0 Å by micro-electron diffraction (micro-ED) and to 1.4 Å by micro-focus X-ray diffraction (Table 1). HnRAC1 formed needle-like crystals that diffracted both electron and X-ray beams well. Especially for electron diffraction, the highest resolution reached a sub-angstrom of 0.82 Å (Fig. 3a; Supplementary Fig. 7a). The structural models obtained from the two different technologies are identical, yet micro-ED is superior to X-ray diffraction on structure resolution in this case (Fig. 3a; Table 1).

**Table 1:** Statistics of crystallographic data collection and atomic refinement of hnRNPA1 segments

Segment (Sequence)	HnRAC1 (^209^GFGGNDNFG^217^)		Non-RAC (^234^GGGYGGS^240^)
	(6J60)	(5ZGD)	(5ZGL)
**Data collection** ^a^			
Radiation source	Electron	Synchrotron	Synchrotron
Wavelength (Å)	0.0251	0.9791	0.9791
Space group	P2_1_2_1_2_1_	P2_1_2_1_2_1_	P2_1_
Cell dimensions			
*a, b, c* (Å)	5.0, 27.8, 36.5	4.9, 27.3, 35.3	12.0, 10.1, 21.4
*α, β, γ* (°)	90.0, 90.0, 90.0	90.0, 90.0, 90.0	90.0, 100.1, 90.0
Resolution (Å)	0.96 (0.994–0.96)^b^	1.4 (1.40–1.49)	0.95 (0.95–0.97)
*R*_merge_	0.251 (0.741)	0.132 (0.367)	0.075 (0.237)
*I*/*σ*(*I*)	3.45 (1.41)	7.8 (3.0)	11.6 (5.3)
CC_1/2_	98.2 (22.1)	99.0 (83.1)	99.1 (94.6)
Completeness (%)	83.0 (73.0)	89.1(65.6)	83.2 (35.7)
Total reflections	14,361	11,951	16,836
Unique reflections	2935	1676	2754
Redundancy	4.9	7.1	6.1
**Refinement**			
Resolution (Å)	0.96	1.4	0.95
No. of reflections	2935	1676	2754
*R*_work_/*R*_free_	0.252/0.267	0.122/0.159	0.100/0.121
No. of atoms			
Protein	63	63	78
Water	4	4	4
B-factor (Å^2^)			
Protein	5.9	3.4	6.5
Water	19.2	5.2	30.6
R.m.s deviations			
Bond lengths (Å)	0.014	0.007	0.012
Bond angles (°)	1.04	0.802	1.200

^a^Two crystals for hnRAC1 electron diffraction (1 spot per crystal) were used

^b^Values in parentheses are for the highest-resolution shell

The structure revealed a cross-β architecture (or steric zipper^18^) with hydrophilic sheet interface composed by N213 and N215 (Fig. 3b). Hydrophilic interface has been mainly seen in amyloids formed by prion-like domains and LCs, and contributes to fibril instability, in contrast to the hydrophobic interface that is abundant in irreversible pathological fibrils^18,19,24,28^ (Supplementary Fig. 7b). Moreover, the hnRAC1 structure exhibits a distinct feature of negatively charged D214 continuously stacking along the parallel in-register β-sheets (Fig. 3b). Stacking-D engenders instability to the fibril architecture due to electrostatic repulsion and hence is barely seen in pathological fibrils (Supplementary Fig. 8). Note that in the local chemical environment of the hnRAC1 crystal, D214 is in contact with the amino terminus of hnRAC1. To evaluate whether this contact exists in fibrils and whether the behavior of D214 is relevant to that of the full-length protein, we modified the N-terminus of hnRAC1 with acetylation and performed D214 mutagenesis on both hnRAC1 and full-length hnRNPA1. The result showed that N-terminally acetylated hnRAC1 formed reversible amyloid fibrils with a similar diffraction pattern to that of the non-acetylated hnRAC1 by X-ray diffraction (Supplementary Fig. 9), which indicates that the amino terminus of hnRAC1 is dispensable for the fibril structure and reversibility. In contrast, as we mutated D214 to V or N, although the resulted peptides still formed amyloid fibrils with no profound structural change (Supplementary Fig. 10), unlike the WT fibril, the mutant fibrils were stable and did not dissociate as temperature increased (Fig. 3c; and Supplementary Fig. 5). Consistently, D214V mutation also resulted in the irreversible fibril formation of full-length hnRNPA1 (Fig. 3c).

Another structural feature of hnRAC1 structure lies on a sharp kink at G211. Kinked structure has been shown to widely present in various LCs for protein network assembly^23,30^. Similarly, in the hnRAC1 structure, kink at G211 enables the aromatic ring of F210 to reach out and interact with the Phe residues from neighboring molecules via π–π stacking (Fig. 3d). The intermolecular π–π interactions may cross-link single fibrils to form hydrogels. To further assess the role of F210 and F216, we mutated them to Ala, respectively. The result showed that the mutations impaired hydrogel formation of hnRNPA1, whereas did not significantly affect the formation of reversible amyloid fibrils (Fig. 3e). In addition, we mutated G211 to Val to constrain the geometry of F210. Similar to the result of F210 mutation, G211 mutation failed in gelation, while retained the ability of reversible amyloid formation (Fig. 3e). Thus, (G)F(G) motif is important for inter-fibrillar interactions and may also contribute to recruiting other molecules into stress granules.

As a control, we also determined the atomic structure of segment ^234^GGGYGGS^240^ by X-ray diffraction (Table 1). This segment does not form fibrils or hydrogels and thus may not be an amyloid core (Supplementary Fig. 4b). Indeed, the structure showed an unfolded conformation with no fibrillar packing (Supplementary Fig. 4b).

### Identification of hnRAC2 and hnRAC3 in hnRNPA1

**Fig. 4:**
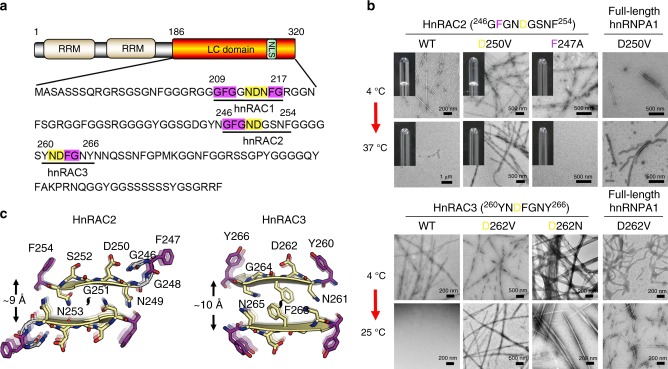
Identification and characterization of hnRNPA1 RAC2 and RAC3. **a** HnRAC2 and hnRAC3 contain the characteristic (N)D(N) (highlighted in yellow) and (G)F/Y(G) (highlighted in magenta) motifs identified in hnRAC1. RRM: RNA-recognition motif. NLS nuclear localization sequence. **b** Amyloid fibril and/or hydrogel formation of hnRAC2, hnRAC3 and full-length hnRNPA1 mutants. Amyloid fibrils were imaged by TEM. Reversibility of amyloid fibrils and hydrogels was monitored as temperature increased from 4 °C to 25 °C or 37 °C. **c** Structures of hnRAC2 and hnRAC3 fibril spines modeled by Rosetta modeling. The fibril core region of each model is highlighted in yellow. The flanking aromatic residues are in purple. The distances between the sheet pairs are indicated. The twofold screw axis is indicated. The fibril axis is perpendicular to the page

According to the structural characteristics of hnRAC1 fibrils, we revisited hnRNPA1 LC for segments that contain (N)D(N) and (G)F/Y(G) motifs. Notably, in addition to hnRAC1, we found another two candidates, ^246^GFGNDGSNF^254^ (named hnRAC2) and ^260^YNDFGNY^266^ (named hnRAC3) (Fig. 4a). We synthesized the two segments and observed that both of them indeed formed reversible fibrils and hydrogels, similar to the behavior of hnRAC1 (Fig. 4b). The reversible fibrils formed by hnRAC2 and hnRAC3 exhibited typical cross-β architectures by X-ray fibril diffraction (Supplementary Fig. 10).

Based on the atomic structure of hnRAC1 and the distance restrains of hnRAC2 and hnRAC3 derived from X-ray fibril diffraction, we built the structural models of hnRAC2 and hnRAC3 by Rosetta modeling (Fig. 4c; Supplementary Note 1). The structure of hnRAC2 showed that the side chains of N249 and N253 face inward the sheet interface and the side chain of D250 facing outward and stacking along the fibril axis. Mutation of D250V impaired the reversibility of fibrils formed by both hnRAC2 and full-length hnRNPA1 (Fig. 4b). Similar to that of hnRAC1, the steric zipper of hnRAC2 kinked at G248, which enables F247 to reach out for intra-fibrillar interactions. Indeed, mutation of F247A inhibited hnRAC2 to form hydrogels with no significant effect on the formation of reversible fibrils (Fig. 4b). As well, the N-terminal acetylation showed no influence on the structure and reversible fibril formation of hnRAC2 (Supplementary Fig. 9).

HnRAC3 fibril spine features a similar architecture to those of hnRAC1 and hnRAC2. The sheet interface is composed of the side chains of N261, F263, and N265 (Fig. 4c). The side chain of D262 facing outward the sheet interface presenting as a signature stacking-D. Importantly, mutations of D262V and D262N, which have been identified in familial ALS patients^12^, resulted in both hnRAC3 and full-length hnRNPA1 to form irreversible fibrils that were not able to disassemble as temperature increased (Fig. 4b). Taken together, our structural studies reveal a key role of stacking-D in the reversibility of amyloid formation, and explain that the disease mutations on the Asp residues may impair the reversibility of hnRNPA1 fibrils and hence enhance irreversible amyloid aggregation and pathogenesis of ALS.

In addition, we surveyed the LC domains of other members in the hnRNP family, including hnRNPA2, hnRNPDL, hnRNPH, hnRNPK, and hnRNPR, which were identified to be closely associated with different diseases, such as ALS, spinal muscular atrophy (SMA), and cancer^31–34^. We found several potential RAC segments and further characterized their capability of reversible amyloid fibril formation. As a result, for each protein, we were able to identify RAC segments that can form reversible amyloid fibrils (Supplementary Fig. 11). Notably, the sequence of hnRAC3 in hnRNPA1 also exists in hnRNPA2, and even more, mutation of Asp in this segment of hnRNPA2 has also been identified in ALS familial mutations^12^. This result indicates that RACs with stacking-D are widely adopted by proteins in the hnRNP family for the formation of reversible amyloid fibrils.

### The function of hnRNPA1 reversible amyloid formation

**Fig. 5:**
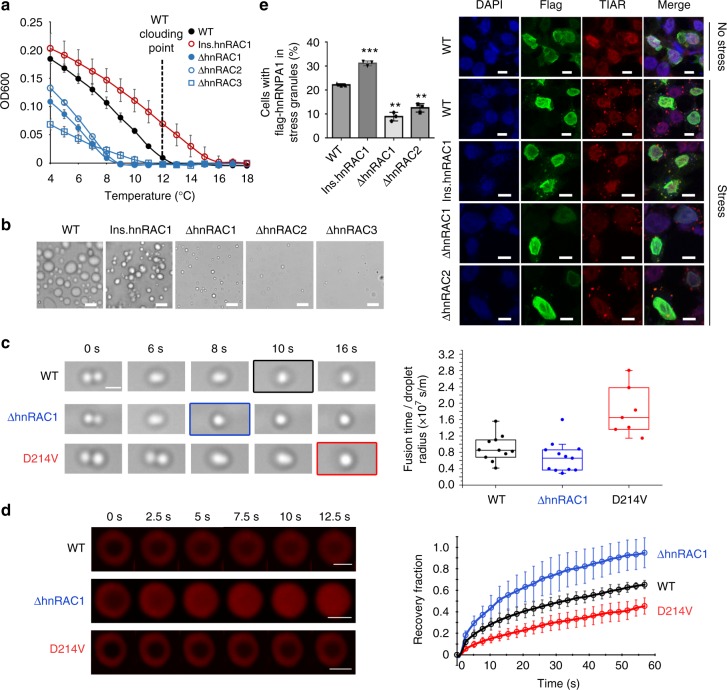
The role of reversible amyloid formation in hnRNPA1 phase separation and stress-granule assembly. **a** Phase diagram measurements of hnRNPA1 variants. The dashed line indicates the clouding point of WT (the temperature at which droplets emerge). Data shown are means ± s.d., with *n* = 3 individual experiments. WT wild type, ins insertion, Δ deletion. Source data are provided as a Source Data file. **b** Representative DIC images of liquid-like droplets formed by hnRNPA1 variants at 6 °C. Scale bars are 10 μm. **c** Effects of reversible amyloid formation on the fluidity of liquid-like droplets measured by optical tweezers. Montages show representative processes of droplet fusion. The boxplot shows the fusion time of droplets divided by mean drop radius. The line within the boxplot indicates the median. Edges of the boxes are the 25th and 75th percentiles. The whiskers extend to outliers. Source data are provided as a Source Data file. **d** Effects of reversible fibril formation on the mobility of liquid-like droplets by FRAP. Montages show the processes of droplet recovery after photobleaching. The graph on right shows the recovery fraction as the function of time. Data shown are means ± s.d., with *n* = 5 individual droplets. The time point right after photobleaching is set to 0. Source data are provided as a Source Data file. Scale bars are 2.5 μm. **e** Recruitment of hnRNPA1 variants into stress granules. DAPI stains the nucleus. HnRNPA1 fused with a Flag-tag was overexpressed in HeLa cells and localized in the nucleus. In response to stress, hnRNPA1 was released to the cytosol and involved in stress granules. TIAR is a marker protein for stress granules. Stress was applied by addition of 0.5 mM sodium arsenate. Scale bars are 10 μm. The bar graph on left shows the percentage of cells with flag-tagged hnRNPA1 in stress granules. Approximately 300 cells from 15 images were calculated for each variant. Errors are s.d. of three individual experiments. **p-value < 0.01; ***p-value < 0.001 calculated by Student’s *t* test. Source data are provided as a Source Data file

To assess the role of reversible amyloid formation in phase separation and the involvement of stress-granule assembly of hnRNPA1, we first examined the influence of hnRACs to the phase-separation diagram of hnRNPA1. We deleted each individual RAC from the full-length hnRNPA1, respectively. Phase separation diagrams of the variants showed a significant deficiency in phase separation with lower clouding points and smaller amounts and sizes of droplets (Fig. 5a, b). As a control, deletion of ^234^GGGYGGS^240^ showed no significant effect (Supplementary Figs. 4b, 12). Reversely, we inserted an additional hnRAC1 into hnRNPA1 at the position between residues 198 and 199. This variant showed a significant promotion of phase separation with an increase of clouding point from 12 °C to 15 °C (Fig. 5a, b).

To assess the effect of amyloid formation on the physical property of the droplets, we next measured the fusion time of hnRNPA1 droplets by optical tweezers. The data showed that hnRAC1 deletion caused the droplets to fuse more quickly than that of the wild-type (Fig. 5c). In contrast, D214V mutation that promotes irreversible amyloid formation, caused the droplets to take longer to fuse (Fig. 5c). Then, we monitored the fluorescence recovery after photobleaching (FRAP) of the droplets. We photobleached a small portion in the center of a droplet and observed that the fluorescence signal of the droplets formed by hnRNPA1 with hnRAC1 deletion, recovered more quickly than that of the wild-type, while D214 mutation resulted in a slower recovery than that of the wild-type (Fig. 5d). Taken together, these data indicate that reversible amyloid formation decreases the fluidity and mobility of hnRNPA1 droplets, and hence reinforces the phase separation.

We next investigated the role of hnRACs in the assembly of stress granules in vivo. We observed that in response to stress, Flag-hnRNPA1 was released from the nuclei to the cytosol and recruited into stress granules (Fig. 5e). Deletion of either hnRAC1 or hnRAC2 significantly diminished the recruitment of hnRNPA1 into stress granules, while insertion of hnRAC1 reversely enhanced it (Fig. 5e). Note that deletion of hnRAC3 is not applicable for this experiment since it resulted in leaking of hnRAC3 from the nucleus to the cytosol when there is no stress due to a potential overlap of hnRAC3 with the nuclear localization sequence (NLS)^35^ (Supplementary Fig. 13). The effects of hnRACs on stress granule assembly in vivo is consistent with that observed on hnRNPA1 phase separation in vitro, which together provide evidence for the important functional role of reversible amyloid formation in hnRNPA1 phase separation and stress-granule formation.

## Discussion

In this work, we observed a synchronous formation and deformation of hnRNPA1 droplets and reversible fibrils (Fig. 1), which indicate that the reversible fibrils may play a role in hnRNPA1 phase separation. By optical tweezers and FRAP experiments, we show that reversible fibrils solidify the liquid-like droplets, which by changing the mechanical property of the droplets, reinforces hnRNPA1 phase separation (Fig. 5c, d). Thus, our work suggests that reversible fibrils play an important functional role in stress granule assembly. From a physical view, membrane-less assemblies are condensed matter of biological macromolecules. Amyloid fibrils as super-molecular polymers could confer such condensed matter with a property of complex fluid that features plasticity and sensitivity to the microenvironmental change and molecular signals. Indeed, it has been reported that stress granules and nuclear paraspeckles consist of stable substructures with a hard core and a mobile shell, which indicates a complex state of molecular assembly with manifold interactions besides low-affinity multivalent interactions^36–38^. To this end, study of the physical and chemical properties of reversible fibrils and the transition of different assemblies of hnRNPA1 and others (e.g., FUS) would help to understand the hierarchical assemblies of membrane-less organelles and biological regulations to this process.

In addition, we also observed the influence of hnRAC deletions on the initiation of phase separation (Fig. 5a). This might reflect a mixed effect of both valence decrease and deficient fibril formation. Weak multivalent interactions are known to drive the phase separation of proteins^39^. While, amyloid fibrils may strengthen the low-affinity interactions by condensing valences^40–43^, and thus may enhance phase separation.

**Fig. 6:**
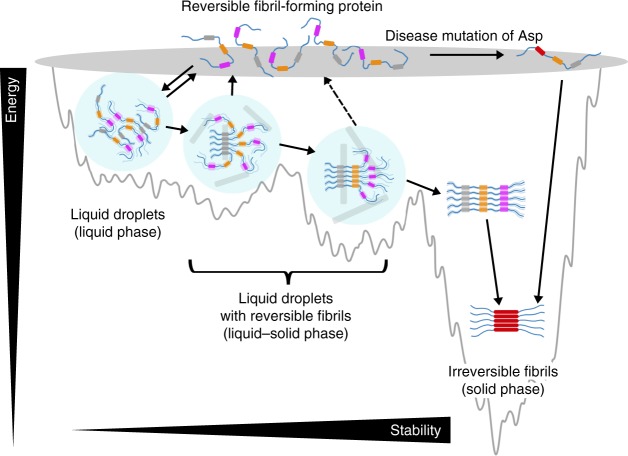
Schematic illustration of hnRNPA1 phase transition. Reversible amyloid-forming proteins such as hnRNPA1 contain multiple RACs that may be gradually involved in the fibril spine as maturation. The less RACs involved in the fibril spine, the more reversible the fibrils would be. Disease mutation of Asp promotes the formation of irreversible fibrils. RACs are shown as gray, orange, and magenta bars. Reversible fibrils are shown as light gray sticks. Light blue solid circles indicate droplets. Irreversible amyloid-forming cores are shown as red bars

Amyloid fibrils as highly ordered protein super-molecular polymers are commonly thermostable and pathological that are related to numerous neurodegenerative diseases^17,19,44^. RNA-binding proteins, such as FUS, hnRNPA1, and TDP43, contain LC domains that are prone to form pathological amyloid fibrils associated with diseases, such as ALS^6,10,45^. While, recent studies have shown that LCs can also form metastable or reversible amyloid fibrils^20–24^. Thus, it is elusive how LCs form pathological irreversible amyloid fibrils as well as dynamic reversible fibrils, what is the structural difference between these two fibrillar assemblies, and what regulates their formation. In this work, we observed the state transition of hnRNPA1 regulated by temperature from dynamic liquid–solid co-existing phase to solid irreversible fibrils. Liquid droplets and reversible fibrils are dynamic states of protein assemblies with high free energies, whereas irreversible fibrils are more thermostable (Fig. 6). It appears that irreversible amyloid-forming proteins commonly use a continuous region to form amyloid fibril spine^46,47^ (Supplementary Fig. 8). In contrast, reversible amyloid-forming proteins, e.g., FUS and hnRNPA1, contain multiple amyloid-forming cores^24^. For example, hnRNPA1 contains three RAC segments that are separated by highly flexible R/G-rich regions (Fig. 4a), and thus it is conceivable that amyloid fibrils may form heterogeneously via different RACs and different numbers of RACs. As more RACs are involved in the fibril spine, the fibrils might turn more stable and difficult to reverse (Fig. 6). Indeed, we observed that deletion of hnRACs, especially hnRAC3, obliterated irreversible fibril formation (Supplementary Fig. 14). Certainly, the irreversible amyloid core, if exists, may also contribute to the irreversible amyloid formation. Moreover, once a reversible amyloid core lost its reversibility due to mutation, irreversible fibril formation could be largely promoted, which provides mechanistic explanation for the pathological condition of Asp mutations identified in familial ALS^12^ (Fig. 6). Therefore, these evidence suggest that reversible amyloid fibrils of hnRNPA1 are dynamic intermediates en route to irreversible fibrils, which is reminiscent of amyloid oligomers that are pathological intermediates of irreversible fibrils^48–50^, while reversible fibrils serve as a functional intermediate. To benefit from the reversible fibril formation and meanwhile encounter the risk of irreversible fibril formation, cells need to employ tight regulation on protein-phase separation, failure of which may lead to diseases.

Our previous work on RAC segments of FUS LC showed the structural mechanisms of two reversible amyloid fibril spines^24^. One exhibits an ordered-coil architecture distinct from the cross-β structure that usually seen in amyloid fibrils; the other features a hydrous sheet interface that is different from the dry interface of known amyloid fibrils (Supplementary Fig. 15). In this work, we present another different structural mechanism for reversible amyloid fibrils formed by RAC segments of hnRNPA1, which features stacking-D in the fibril core (Supplementary Fig. 15). A recent NMR study also suggests the involvement of D290 in destabilizing the self-association of hnRNPA2 LC domain^51^. In addition, kinked β-sheets (so called LARK) have also been suggested to destabilize fibril formation^23,34^. Taken together, it is plausible to demonstrate that structural diversity is an essential difference between reversible and irreversible amyloid fibrils. There are various mechanisms allowing reversibility, while irreversible fibrils commonly adopt a uniform structure of steric zipper with dry (usually hydrophobic) interface^18,19,28^. Steric zipper is generally a highly thermostable architecture of protein super-molecular assembly, whereas, many disturbances, such as hydrophilic and/or hydrous sheet interface, ordered-coil and kinked β-sheet (LARK) configurations, and stacking-D in the fibril core may introduce instability to amyloid fibrils and generate reversible amyloid fibrils. More importantly, these structural features provide bases for regulation of dynamic amyloid assembly, disruption of which thus may lead to pathogenesis of neurodegenerative diseases.

## Methods

### Protein expression and purification

Wild-type hnRNPA1 in vector pET-9d was purchased from Addgene (Plasmid #23026). Variants of hnRNPA1 were subcloned from the wild-type construct. Recombinant hnRNPA1 and variants were overexpressed in *E. coli* BL21(DE3) pLysS with the addition of 0.4 mM IPTG at OD_600_ = 1.0–1.2 and further incubation at 25 °C overnight. Cells, except for those expressing the D262V mutant, were harvested and lysed in buffer containing 50 mM Tris-HCl pH 7.5, 100 μg ml^−1^ RNAase (Roche), 2 mM DTT and 1 mM PMSF. The lysate was centrifuged at 30,966 *g* at 4 °C. The supernatant was loaded onto a 5-ml SP FF column connected with ÄKTA Purifier (GE Healthcare, USA). The proteins were eluted with a gradient mixing of buffer A (50 mM Tris-HCl pH 7.5 and 2 mM DTT) and buffer B (buffer A with 1 M NaCl). Fractions were analyzed by SDS-PAGE. Those mainly containing hnRNPA1 were collected and further purified by Superdex 75 16/60 column (GE Healthcare) pre-equilibrated with buffer containing 50 mM Tris-HCl pH 7.5, 500 mM NaCl, and 2 mM DTT. Fractions containing hnRNPA1 monomers were collected and concentrated. The D262V mutant was overexpressed and purified with the same procedure as the others, except that the buffer pH was adjusted to Bis-Tris pH 6.5. All purified proteins were changed to buffer containing 50 mM Tris-HCl pH 7.5, 100 mM NaCl for the following experiments.

The FUS RGG region (residues 371–526) was constructed in vector pET-28a and overexpressed in *E. coli* BL21(DE3) by induction at OD_600_ = 1.0–1.2 with 0.5 mM IPTG and further incubation for 24 h at 16 °C. Cells were harvested and lysed in buffer containing 50 mM Tris-HCl pH 8.0 and 6 M guanidine hydrochloride. The cell lysate was then centrifuged at 30,966 *g* for 1 h. The supernatant was loaded onto a 5-ml Ni column (GE Healthcare, USA). The bound proteins were eluted with buffer solution containing 50 mM Tris-HCl pH 8.0, 6 M guanidine hydrochloride, and 50 mM imidazole. The eluted proteins were further purified by HPLC (Agilent, USA) with the elution buffer containing 35% v/v acetonitrile. The purified protein was freeze-dried by FreeZone lyophilizers (Thermo, USA) and stored at −20 °C.

### DIC microscopy

In total, 100 μM hnRNPA1 wild-type and variants were incubated at 6 °C and LLPS occurred in minutes. In all, 3 μl of protein samples were dropped on a glass slide. The slide was placed on a TP-CHSQ-C thermal stage that was maintained at 6 °C and checked by a Leica SP8 confocal microscope. Images were taken by a DMI8 camera with a 63 × 0.7 NA dry objective.

### Negative-staining TEM

To observe the content of droplets, 80 μM hnRNPA1 was placed on ice for 20 min. In total, 4 μl of the sample was transferred to a glow-discharged TEM grid (Cu, 300 mesh; Beijing Zhongjingkeyi Technology Co., Ltd.). The grid was washed with 4 μl pre-cooled 3% w/v uranyl acetate, and then stained by 4 μl pre-cooled 3% w/v uranyl acetate for 45 s. During sample preparation, most droplets were washed off the grid or disrupted by uranyl acetate solution. To observe an integrate droplet, multiple grids were prepared in parallel and carefully checked by TEM. Since the thickness of droplet weakens electron transmission and blurs the imaging of fibrils inside, we also prepared grids with an additional step of washing with 4 μl pre-cooled ddH_2_O before staining to fully disrupt the droplets. Before acquiring TEM images, the excess buffer on grids was removed and dried in air. Grids were loaded on a FEI Tecnai T12 TEM working at 120 KV. The TEM is equipped with LaB_6_ filament and BM-eagle camera with the pixel of 4096 × 4096. Exposure time for each image was 2 s.

Amyloid fibril sample grids were prepared and observed by TEM following the same protocol. To check the reversibility of fibrils, the fibril samples were incubated at 25 °C for 20 min and then observed by TEM.

### ThT fluorescence staining

Overall, 19 μl of 100 μM hnRNPA1 were placed on ice. The protein solution turned cloudy in seconds, followed by addition of 1 μl of 1 mM ThT and further incubation for 5 min. The sample was observed at 4 °C by a Leica SP8 confocal microscope equipped with a 458 laser and a TP-CHSQ-C thermal stage. ThT signals were detected by a HyD detector. To test the fibril reversibility, the stage temperature was gradually increased from 4 °C to 16 °C and further to 25 °C. The sample was incubated at each temperature for 2 min. The control sample of the FUS RGG region was prepared and observed following the same protocol.

### Amyloid fibril formation

Reversible amyloid fibrils of full-length hnRNPA1 were observed as phase separation by placing 80 μM hnRNPA1 on ice for 20 min. To test the mutation of Asp residues to the reversibility of the full-length hnRNPA1 fibrils, hnRNPA1 proteins with mutations at the concentration of 100 μM were placed on ice for 20 min to form fibrils. The fibril reversibility was assessed by increasing the temperature from 4 °C to 25/37 °C.

Peptides were synthesized with free termini or N-terminal acetylation as indicated by ChinaPeptides Co., Ltd. All peptides were dissolved in ddH_2_O. The peptide sequences and conditions for gelation and fibrillation were listed in Supplementary Table 1. The reversibility was measured by increasing the temperature from 4 °C to 25 °C or 37 °C as indicated in figures for 20 min.

### AFM

Liquid cell and freshly cleaved mica were pre-cooled at 4 °C. Overall, 30 μl of fibril samples were added into the liquid cell. Images were acquired in fluid using a MultiMode AFM instruments with NanoScope V system (Bruker). Measurements were carried out using SNL-10 silicon cantilevers with a spring constant of 0.24 N m^−1^ (Bruker) in scanasyst mode. Disassembly of fibrils was monitored by AFM with a scan rate of 0.9 Hz upon temperature increase from 4 °C to room temperature. All images were analyzed by NanoScope Analysis 1.5 (Bruker).

### Crystallization and data collection

HnRAC1 was dissolved in ddH_2_O to 3 mg ml^−1^. Crystals were obtained by hanging drop in buffer containing 2.0 M ammonium sulfate, 0.1 M CAPS pH 10.5, 0.2 M lithium sulfate at 16 °C. Peptide ^234^GGGYGGS^240^ was dissolved in ddH_2_O to 20 mg ml^−1^. The crystals were obtained from the buffer containing 0.2 M ammonium sulfate, 30% PEG 4000 at 16 °C by hanging drop.

For Micro-ED data collection, the crystal drop was transferred to a freshly glow-discharged quantifoil EM grid (R2/2, Cu 300 mesh; QUANTIFOIL), followed by blotting from the bottom and washing twice with 5% v/v PEG 200. Then, the grid was vitrified by plunging into liquid ethane using Vitrobot Mark IV (FEI). The grid was then transferred using a Gatan 626 cryo-holder to FEI Tecnai F20 equipped with field-emission gun operating at 200 kV (wavelength = 0.0251 Å). Diffraction images were recorded using Gatan US4000 (895) camera with a sensor size of 4096 × 4096 pixels. The eTasED software (developed by Dr. Xueming Li and available at http://github.com/THUEM/eTasED) was used to perform the semi-automated Micro-ED data collection. The nominal camera length was set to 520 mm, and calibrated by using an oriented-gold-film standard sample. Exposure time for each image frame was 4.7 s. Selected area aperture (10 μm in diameter) was used to limit the sample area for diffraction. The measured electron dose exposed on the sample is ~0.008 e^−^ Å^−2^ s^−1^. During the exposure, crystals were continuously rotated with a speed of 0.28° per second. Therefore, each image frame covered 1.4° wedge, and each dataset from a single crystal covered a total 40°–80° angle range in reciprocal space. XDS was used to index the diffraction data^52^. Datasets from four crystals were merged together and scaled by XSCALE^52^ (Table 1).

For X-ray diffraction data collection, the crystals were diffracted on the micro-focus beam line BL-17U at Shanghai Synchrotron Radiation Facility (wavelength = 0.9791 Å). The crystals were cryo-cooled (100 K) during data collection. Data were processed by using XDS^52^ (Table 1). One crystal (one spot per crystal) was used for data collection.

### Structure determination

For hnRAC1, direct method by SHLEXD^53^ was used to determine the phase information from electron diffraction data, and molecular replacement by Phaser^54^ from Phenixprogram package was used to determine the phase information from X-ray data. The initial searching model consists of a geometrically ideal β-strand composed of four alanine residues. For ^234^GGGYGGS^240^, phase information was determined by direct method using SHELXT/L^55,56^. Structure refinement was performed by using phenix.refine^57^. The atomic models were built by COOT^58^. The statistics of structure refinement were reported in Table 1. The structures were illustrated by PyMol (Schorödinger, LLC).

### X-ray fibril diffraction

In total, 5 μl of fibril samples were deposited between two sealed capillaries and allowed fibril samples to align and dry at 4 °C overnight. The aligned fibrils were diffracted by an in-house Rigaku FR-E generator (wavelength 1.5418 Å) equipped with an R-AXIS IV + + imaging plate detector.

### Rosetta modeling

The structural models of hnRNPA1 hnRAC2 and hnRAC3 were built using Rosetta^59^ (Supplementary Note 1). The hnRACs were modeled as a β-sheet. The β-sheets were assembled by exploring all possible arrangements (different steric zipper classes^28^). The assembled fibril structures were then filtered and refined by simultaneously optimizing the rigid-body degree of freedom between symmetrical copies, side chain, and backbone torsions of hnRACs subunits, guided by full-atom Rosetta energy functions^60^. The intra-sheet and inter-sheet distances of hnRACs fibrils obtained from fibril diffraction were input as restrains. The top-ranking models based on Rosetta energy were selected.

### Phase diagram measurement

In total, 100 μl of 100 μM hnRNPA1 samples were loaded into a 0.5 mm Cuvette. To measure the phase diagram, the temperature of the cuvette was gradually decreased from 20 °C to 4 °C at a rate of 1 °C min^−1^. Meanwhile, OD_600_ values were measured every minute during the temperature decrease by using Chirascan™ CD Spectrometer equipped with a temperature controlled device.

### Stress-granule formation and immunofluorescence imaging

HeLa cell (Cell Bank of the Chinese Academy of Sciences, Shanghai, Cat. Lot: SCSP-504) culture was plated on coverslips for 18–24 h, and then transfected with hnRNPA1 plasmids with a pAGGS vector in DMEM by polyjet (SignaGen Laboratories) for 6 h. Forty-eight hours after transfection, 0.5 mM sodium arsenate was incubated with the cell culture for 1 h to induce stress granules. For immunofluorescence imaging, cells were fixed with a mixture of 4% paraformaldehyde and 4% glucose in PBS for 15 min, and permeabilized in 0.15% triton for 15 min. After blocking with 5% BSA, cells were co-incubated with anti-Flag antibody (Sigma-Aldrich, Cat. Lot: F1804) and anti-TIAR antibody (Cell Signaling Technology Cat. Lot: 8509 S) overnight at 4 °C. Secondary antibodies were conjugated to Alexa Fluor 488 (ThermoFisher, Cat. Lot: A-11029) or Alexa Fluor 594 (ThermoFisher, Cat. Lot: A-11012). The antibodies used were 1000 times diluted. Coverslips were mounted using ProLong™ Gold Antifade Mountant with DAPI (ThermoFisher Cat. Lot: P36935). Images were collected on a SP8 Leica microscope with a DMI8 camera and further analyzed by a Leica LAS AF Lite software. The capability of the recruitment of hnRNPA1 into stress granules was measure by calculating the percentage of the cells with Flag-hnRNPA1 in stress granules in the total number of cells with transfected Flag-hnRNPA1. A total number of ~300 hnRNPA1-transfected cells were calculated for each sample.

### Optical tweezers

An optical tweezer microscope C-trap™ from LUMICKS (Amsterdam, The Netherlands) with two steerable traps was used to perform controlled fusion of droplets^16^. Sample chambers were prepared by first applying two thin pieces of double-stick transparent tape (~0.1 mm thick) in parallel within 5 mm separation to a coverslip, followed by placing a glass slide on top of the coverslip. Liquid droplets of hnRNPA1 and variants were formed with 180 μM of proteins at an environmental temperature of 13 °C. These droplets were flowed into the chamber just before data acquisition. A 1064 nm laser with low light intensity (< 0.5 W) was applied to minimize heating. One droplet was held in place by a trap, and the other steerable trap was used to capture other droplets and bring them toward the stationary droplet with a velocity of 0.04 μM s^−1^ until the two droplets surfaces touched. Fusion events were recorded simultaneously with high laser signal (50 kHz) and low video signal (30 Hz). Fusion times were determined via analysis from the laser signal and confirmed with video signal.

### FRAP assay

WT and mutants were labeled with Alexa Fluor 647 carboxylic acid, tris(triethylammonium) salt (ThermoFisher LOT: A33084) following the manufacturer’s instructions. For the FRAP assay, unlabeled 50 μM WT and mutants (25 mM Tris, 50 mM NaCl, 10% PEG 3350, pH 7.5) mixed with fluorophore-labeled proteins at a molar ratio of 50:1 were incubated at room temperature for 20 min. In all, 6 μl of the samples were loaded on an ultra-thin glass bottom dish. FRAP experiments were performed by using a Leica SP8 microscope with a ×100 oil objective and a 633 laser line. Approximately a 2.5 μm in diameter circular region within the droplets in size of 4–6 μm was bleached for 2.5 s with full laser power. Post-bleach images were acquired at 2.5 s per frame. Images were analyzed with the Leica Application Suite X software. For each time point, the intensity was corrected by the intensity of a neighboring unbleached region. Fluorescence recovery fraction was calculated with the formula (*I*_*t*_ − *I*_minimum_)/(*I*_before bleaching_ − *I*_minimum_). *I*_*t*_ is the intensity at individual time points.

### ThT kinetic assay

Overall, 60 μl of samples containing 50 μM hnRNPA1 or variants and 50 μM ThT were shook at 900 rpm at 25 °C. ThT fluorescence was excited at 440 nm, and the emission wavelength of 485 nm was recorded per 5 ms by a Thermo Scientific Varioskan Flash spectrophotometer. Four replicates for each sample were performed.

### Reporting summary

Further information on research design is available in the Nature Research Reporting Summary linked to this article.

## Supplementary information

Supplementary Information

Reporting Summary

Source Data

## Data Availability

The PDB accession code of hnRAC1 solved by Micro-ED is 6J60 and by X-ray diffraction is 5ZGD. The PDB accession code of non-RAC segment GGGYGGS is 5ZGL. The source data underlying Figs. 1d, 5a, 5c–e and Supplementary Figs. 2b, 3, 12, and 14 are provided as a Source Data file. A reporting summary for this article is available as a Supplementary Information file. All other data supporting the findings of this study are available from the corresponding authors on reasonable request.
