# Supplementary material for: Structural basis for reversible amyloids of hnRNPA1 elucidates their role in stress granule assembly
Source: Nat Commun. 2019 May 1;10:2006. doi: 10.1038/s41467-019-09902-7 (PMC6494871; doi:10.1038/s41467-019-09902-7)
Supplement: Supplementary file 1 — Supplementary Information [file 41467_2019_9902_MOESM1_ESM.pdf]

Supplementary Information for

Structural basis for reversible amyloid fibril of hnRNPA1 as a functional structure behind stress granules

Gui, *et al.*

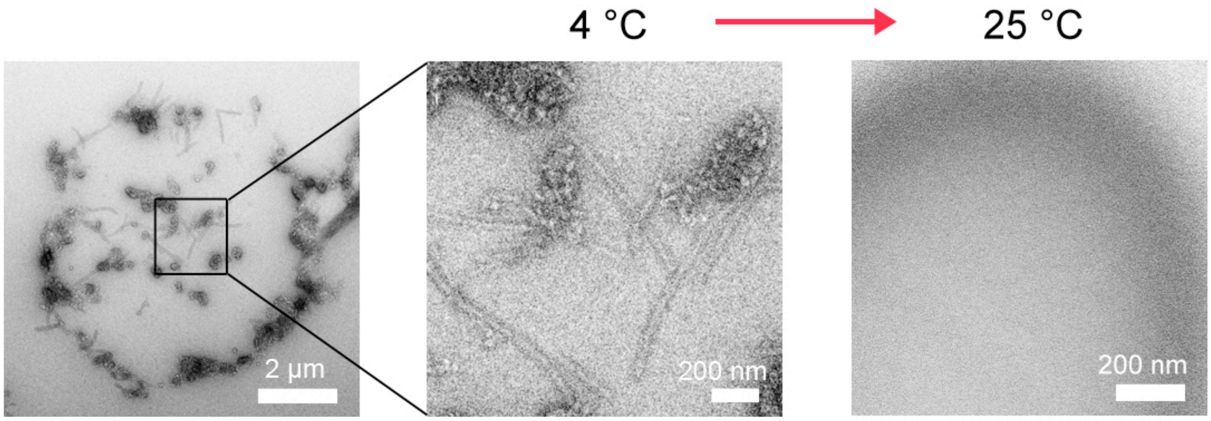

Supplementary Figure 1 TEM images of hnRNPA1 droplets and fibrils. The sample was dropped on an EM grid, rinsed with water, and then stained with 3% w/v uranyl acetate. The surface of the liquid-like droplets collapsed as rinsed with water, yet the fibrils retained. Both droplets and fibrils disappeared as warmed up to 25 °C.

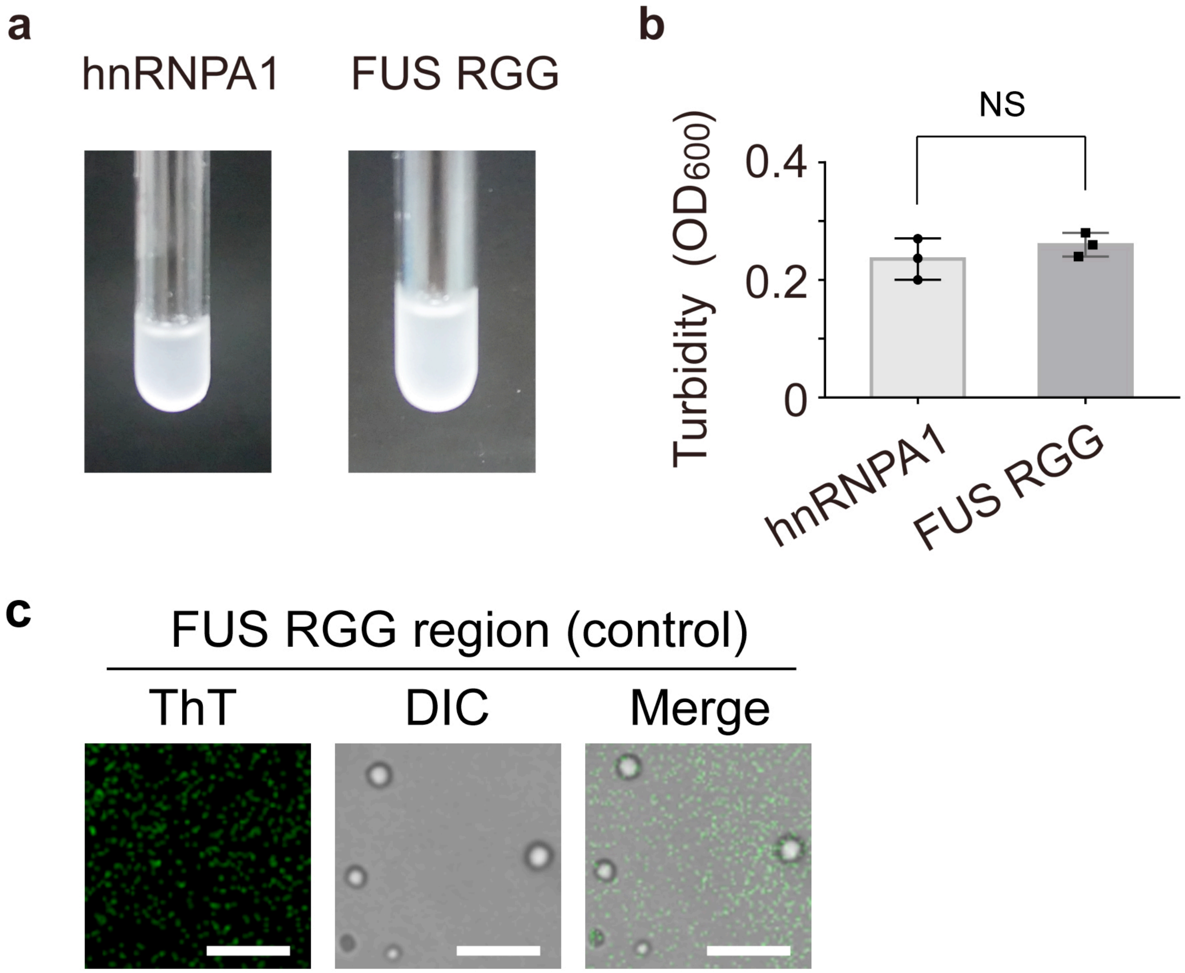

Supplementary Figure 2 Phase separation of hnRNPA1 and FUS RGG region at 4 °C. (**a**) Both solutions became cloudy. (**b**) Turbidity of the solutions in (**a**). Data shown are mean + s.d., with n = 3 independent samples. Values were compared using Student's *t*-test. NS represents non-significant. Source data are provided as a Source Data file. (**c**) *In situ* imaging of the phase separation of FUS RGG region by DIC and fluorescence microscopy as a control of that of hnRNPA1. The fluorescence intensity was boosted by adjusting the gray color depth to visualize ThT. Together with Fig. 1c, the weak and well dispersed ThT intensity in the control sample may rule out the ThT artifact in this experiment and support the fibril formation in the hnRNPA1 sample. Scale bars are 10 μm.

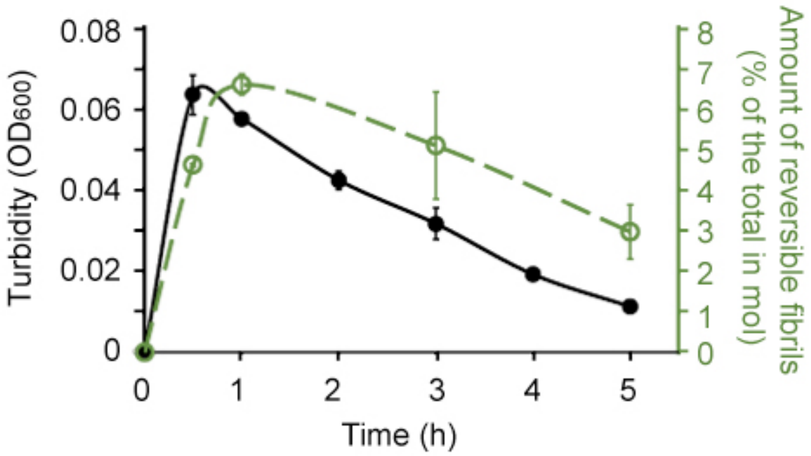

Supplementary Figure 3 Estimation of reversible amyloid fibril amounts (green) and the turbidity of hnRNPA1 solution (black) as functions of time. Data shown are mean ± s.d., with n = 3. The quantity of reversible fibrils were roughly estimated by measuring the ThT fluorescence change before and after the temperature increase from 4 °C to 25 °C. The ThT intensity was calibrated with irreversible fibrils formed at room temperature with an assumption that reversible and irreversible fibrils may have similar ThT fluorescence levels. Source data are provided as a Source Data file.

a

Residue No.	Peptide sequence
208-212/226-230	GGFGG
235-239/303-307	GGYGG
207-213	GGGFGGN
234-240	GGGYGGS
225-231	RGGFGGS
209-217	GFGGNDNFG
221-229	NFSGRGGFG
219-230	GGNFSGRGGFGG
218-226	RGGNFSGRG
224-232	GRGGFGGSR

b

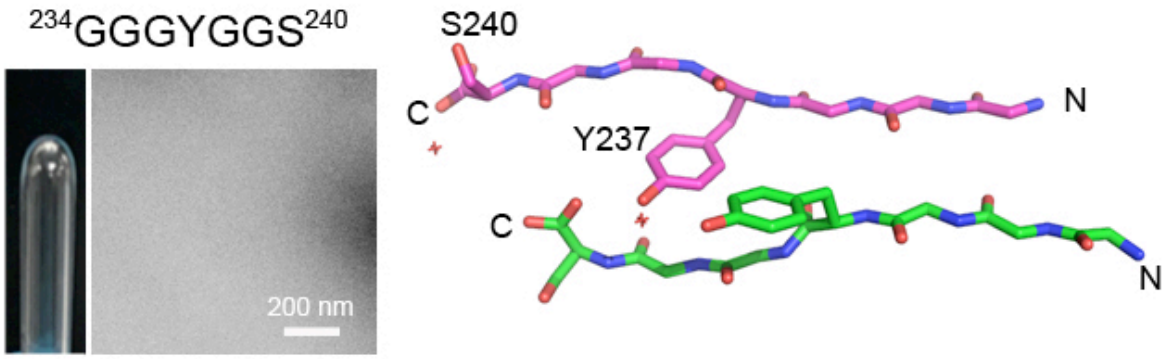

Supplementary Figure 4 Screening for RAC segments from hnRNPA1 LC domain. (**a**) Segments of hnRNPA1 LC selected for screening. HnRAC1 that formed reversible amyloid fibrils, is colored in red. Segment ^{234}GGGYGGS^{240} that was studied as a control is colored in blue. (**b**) Control segment ^{234}GGGYGGS^{240} did not form amyloid fibrils or hydrogels. Consistently, the atomic structure of this segment showed no fibrillar packing.

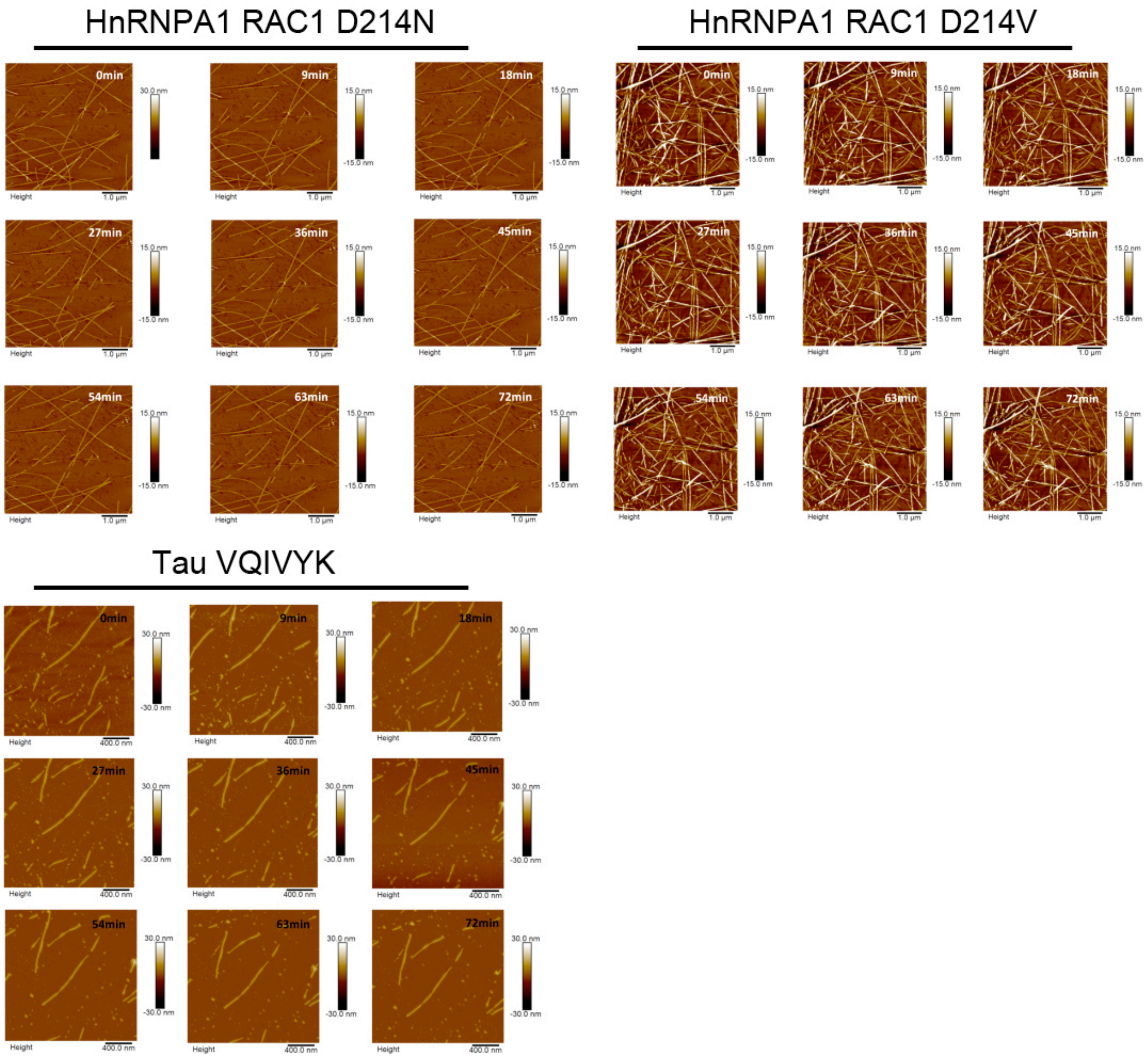

Supplementary Figure 5 Montages of real-time and *in situ* visualization of the amyloid fibrils formed by hnRAC1 mutants and Tau amyloid-forming segment VQIVYK by AFM. The fibrils are irreversible and no change of fibrils was observed in the experiment.

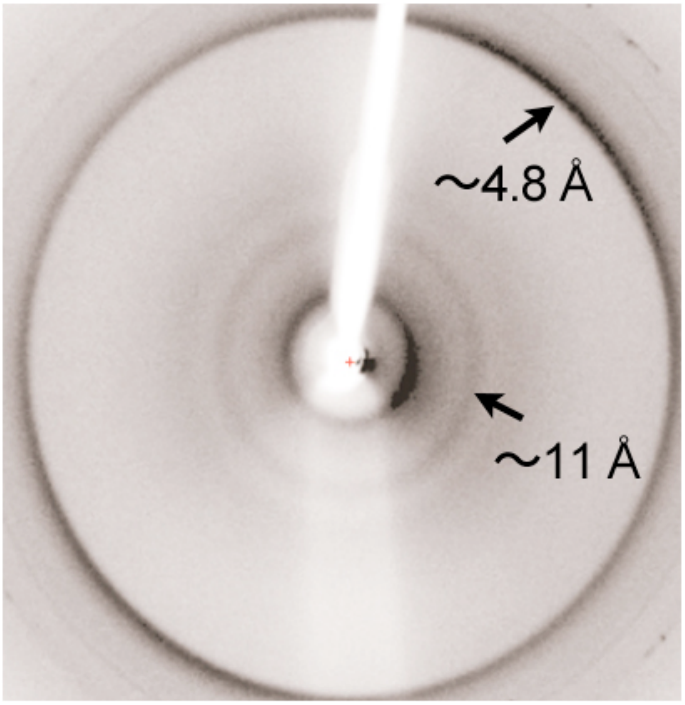

Supplementary Figure 6 X-ray diffraction of hnRAC1 amyloid fibrils.

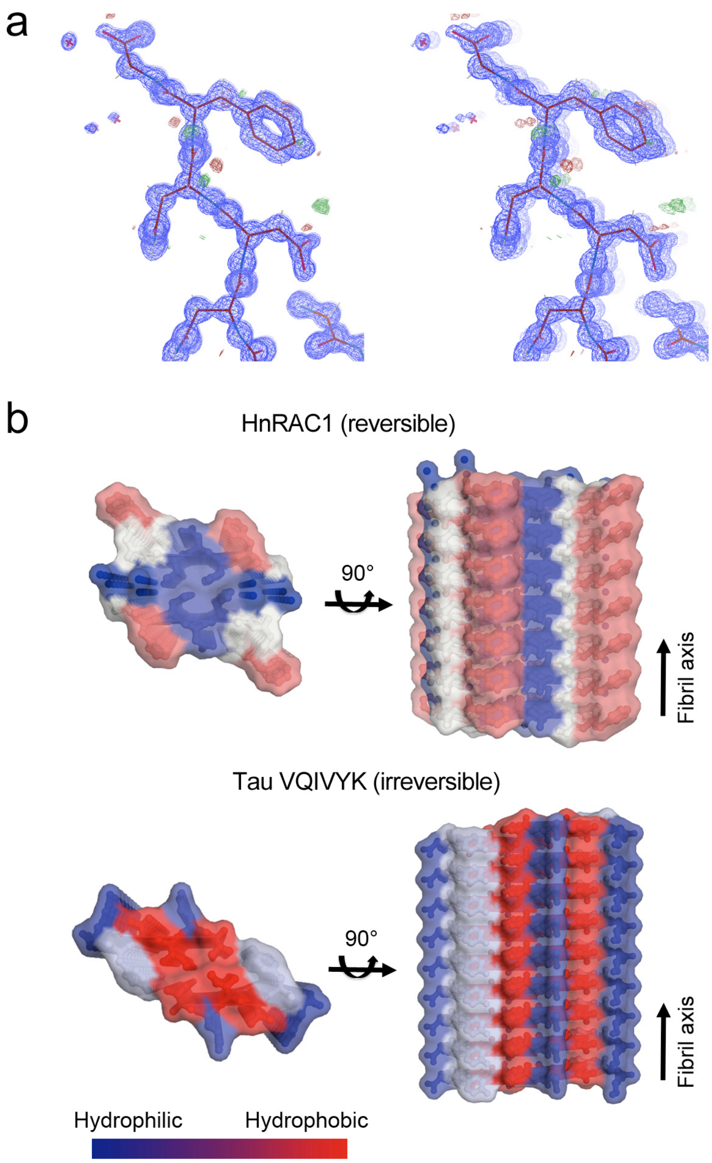

Supplementary Figure 7 Structural analysis of hnRAC1. (**a**) A stereo density map of the micro-ED structure of hnRAC1. 2*Fo*-*Fc* maps are contoured at 1.49 rmsd (blue). *Fo*-*Fc* maps are contoured at 3.23 rmsd (green and red). Red crosses represent water. (**b**) Polarity of amyloid fibril spines of hnRAC1 and Tau VQIVYK. Reversible fibrils formed by hnRAC1 contain a hydrophilic sheet interface. In contrast, irreversible fibrils, such as that formed by VQIVYK, normally contain a hydrophobic sheet interface. PDB IDs: 6J60 for hnRNPA1 RAC1, 2ON9 for Tau VQIVYK.

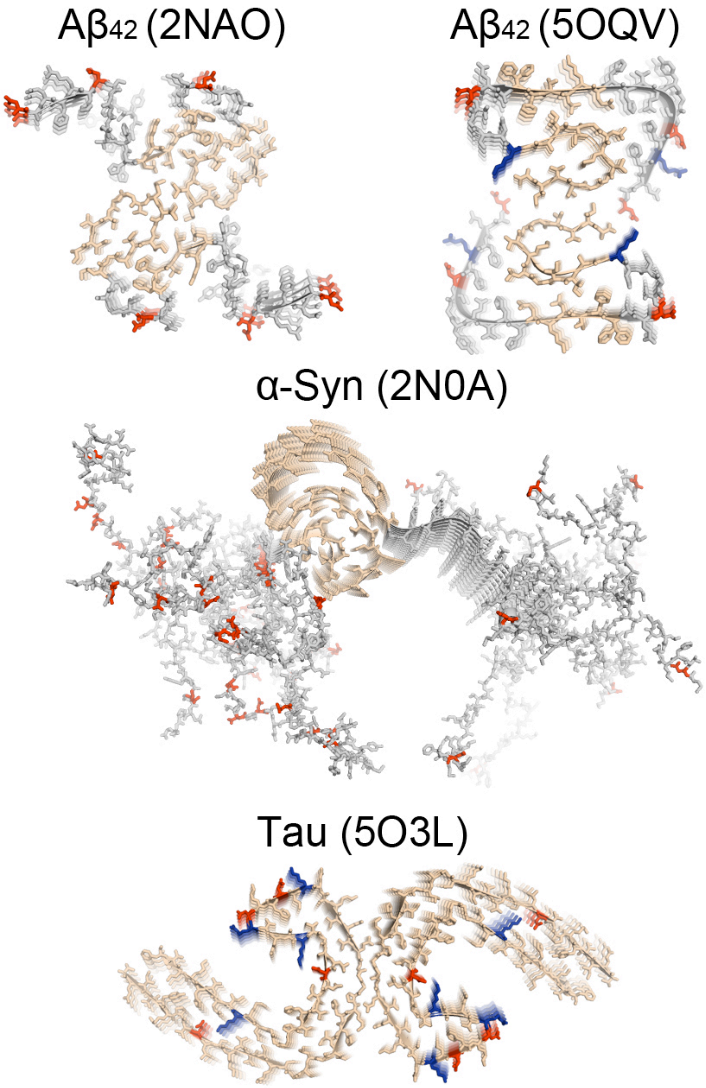

Supplementary Figure 8 Asp residues in various pathological amyloid fibril structures. Asp residues usually locate outside the fibril core regions such as seen in $A\beta_{42}$ and α-syn fibrils. It is also seen in the case of Tau where Asp residues, though involved in fibril core, are neutralized by Lys or Arg. Fibril core regions (in wheat color) are defined according to the steric-zipper-like configurations with interdigitated side chain interactions. Asp residues are in red. Lys and Arg are in blue. Fibril axis is perpendicular to the page. PDB IDs of the fibril structures are indicated in parentheses.

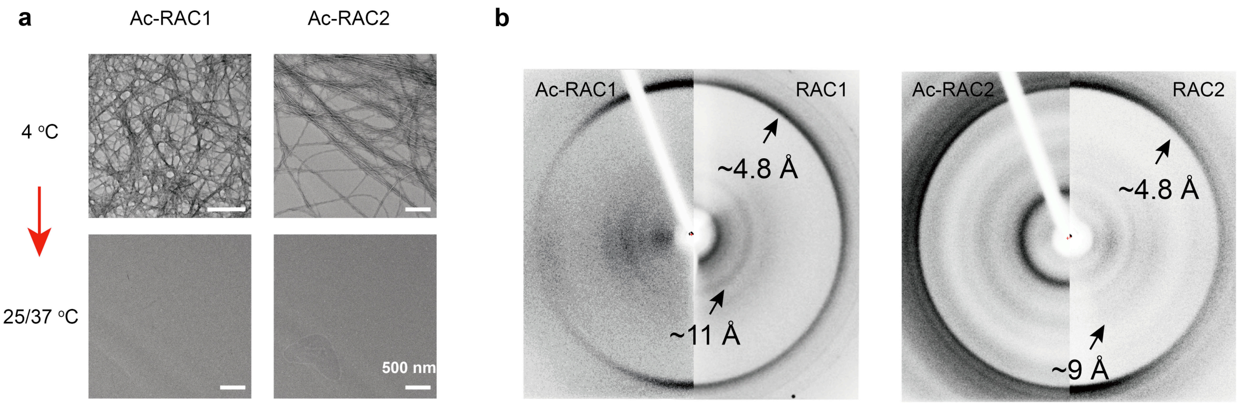

Supplementary Figure 9 Reversible amyloid fibrils formed by N-terminally acetylated hnRACs. (**a**) N-terminally acetylated (abbreviated as Ac) hnRAC1 and hnRAC2 formed amyloid fibrils at 4 °C that dissolved upon temperature increase to 25 °C or 37 °C. Scale bars are 500 nm. (**b**) Comparison of fibril diffraction of Ac-hnRAC1/Ac-hnRAC2 (left-halves) with non-acetylated counterparts (right halves).

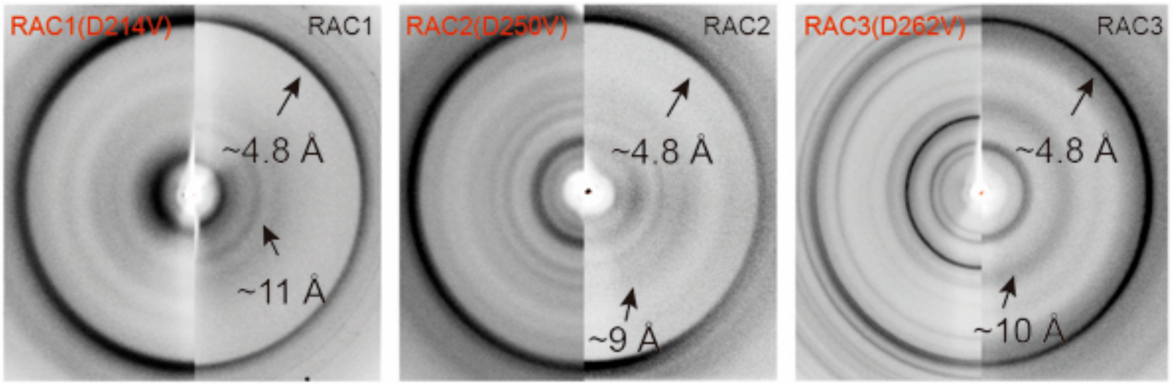

Supplementary Figure 10 Fibril diffraction of hnRAC mutants (left halves) in comparison with the WT hnRACs (right halves). The results showed that D to V mutations did not change diffraction patterns of RAC fibrils.

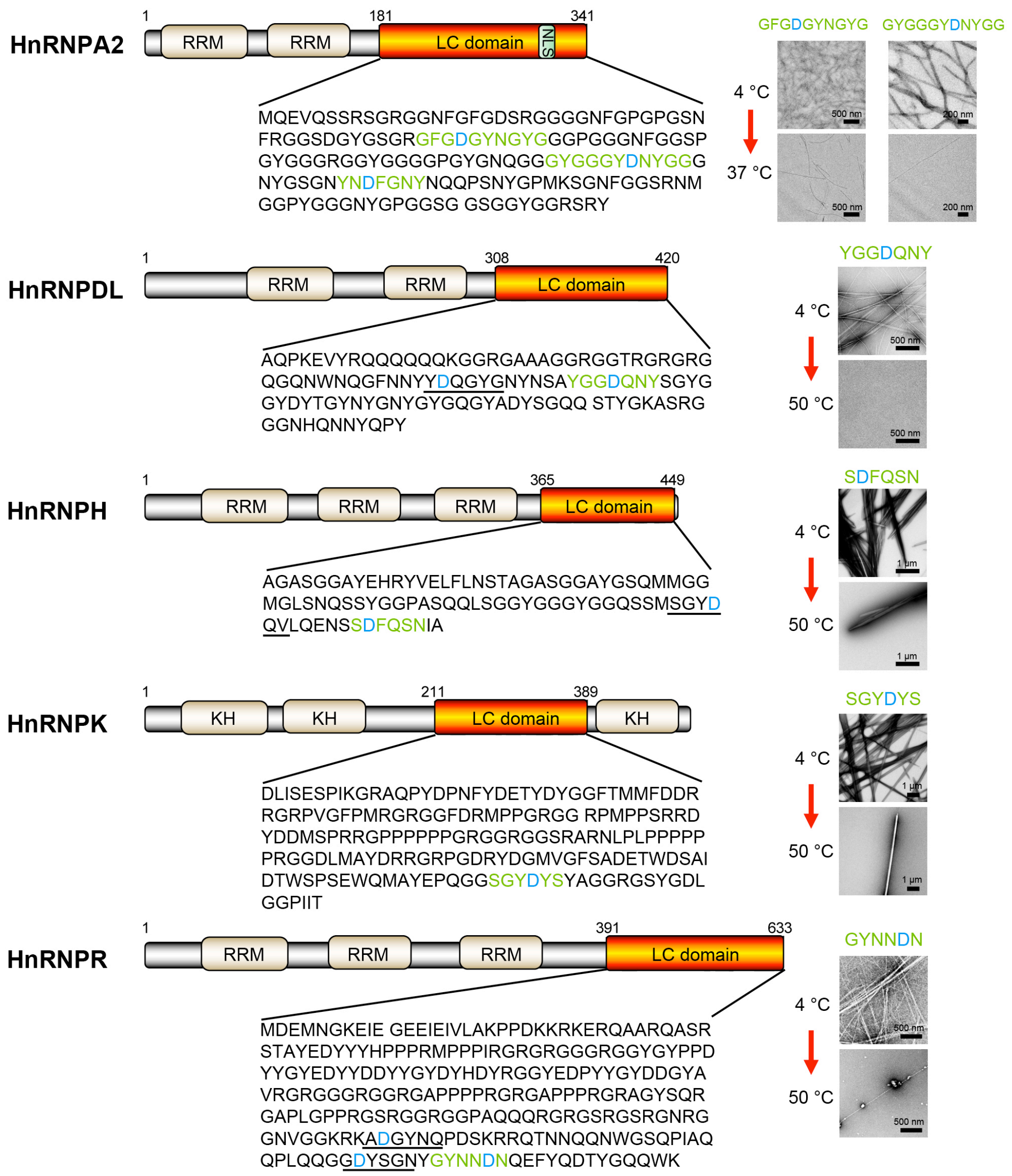

Supplementary Figure 11 Identification of RAC segments from the hnRNP protein family. RAC segments were broadly found in various proteins from the hnRNP family. Amyloid fibrils formed by different RAC segments can be totally or mostly dissembled upon temperature increase. Identified RAC segments are highlight in green. Segments that did not form amyloid fibrils in our tests are underscored. Asp residues in the segments are in blue. Segment YNDFGNY of hnRNPA2 is the same as hnRNPA1 RAC3. RRM: RNA recognition motif. KH: K homology RNA-binding domain.

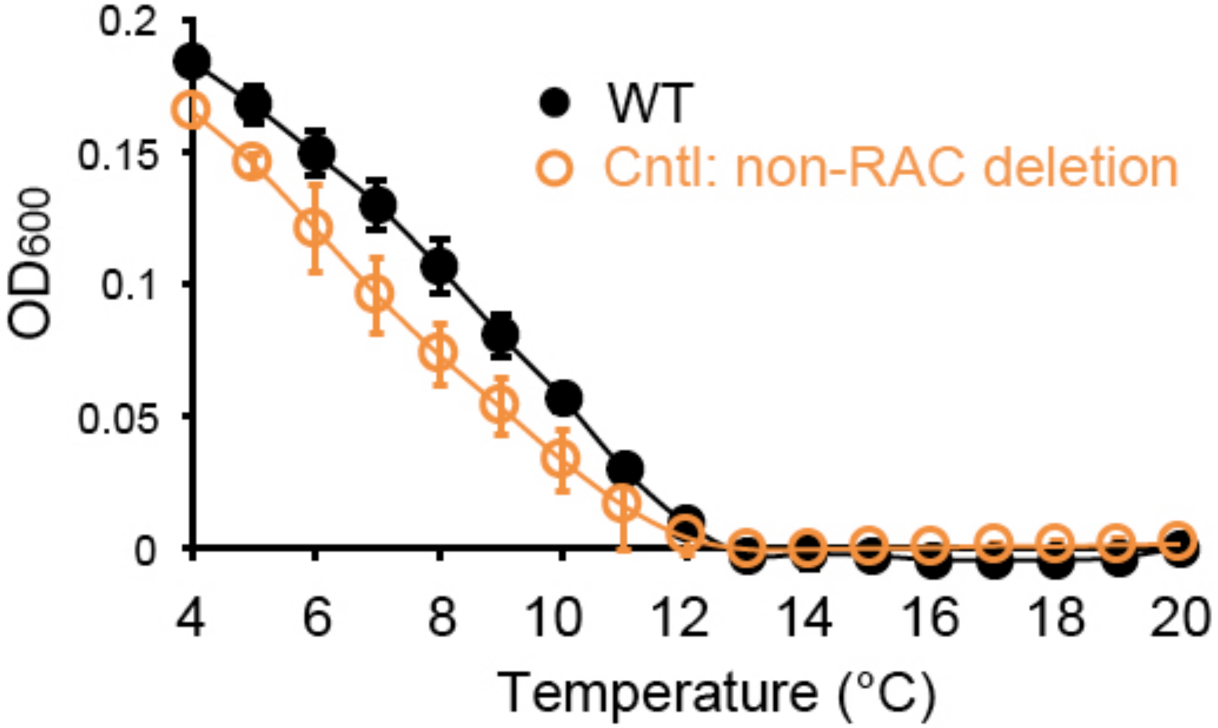

Supplementary Figure 12 Effect of non-RAC segment on the phase diagram of hnRNPA1. Segment ^{234}GGGYGGS^{240} (non-RAC) that did not form amyloid fibrils and hydrogels, was conducted as a control for RAC deletions. Data shown are means ± s.d., with n = 3 individual experiments. Source data are provided as a Source Data file.

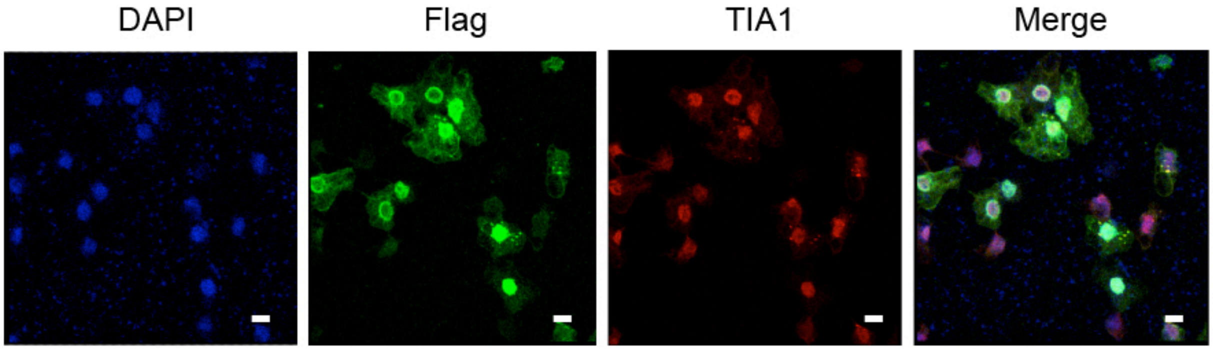

Supplementary Figure 13 Expression of Flag-tagged hnRNPA1 with hnRAC3 deletion in HeLa cells. HnRAC3 deletion resulted in leaking of hnRNPA1 from nucleus to cytosol when there is no stress. Scale bars are 10 μm.

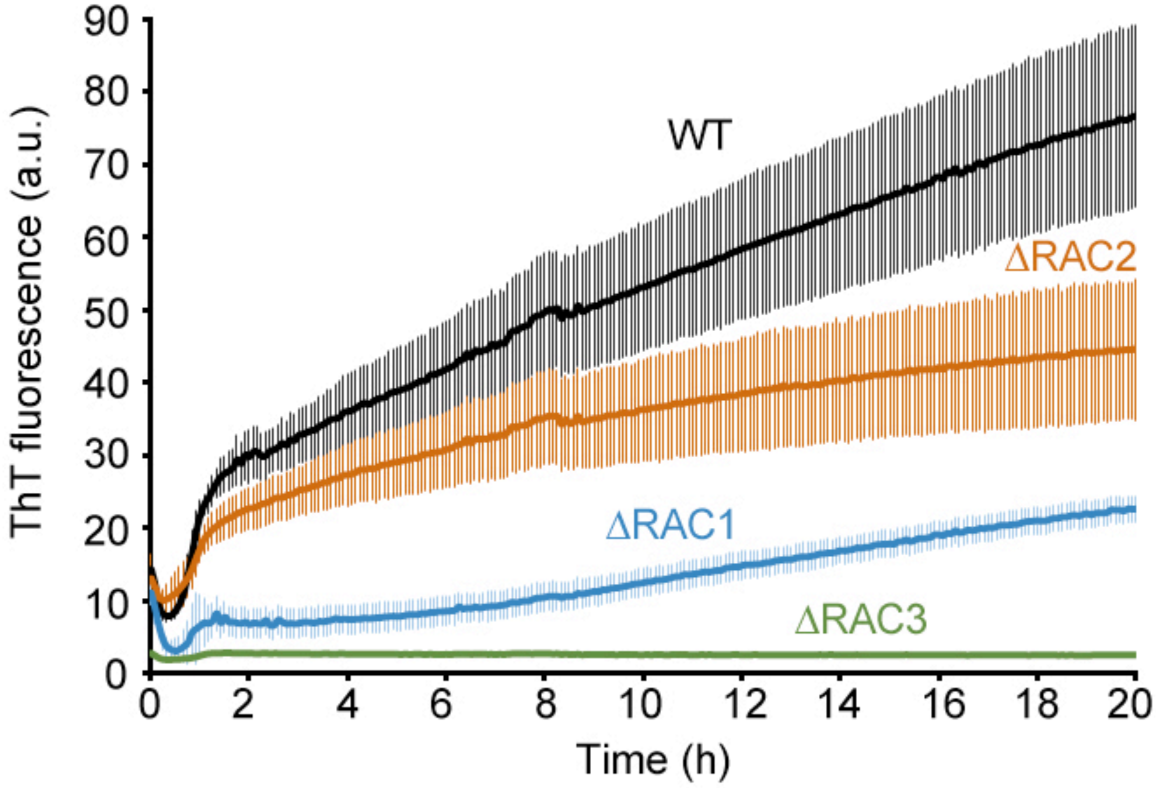

Supplementary Figure 14 Irreversible amyloid fibril formation of hnRNPA1 variants at 25 °C monitored by ThT kinetic assay. Data shown are mean ± s.d., with n = 4 individual samples. Source data are provided as a Source Data file.

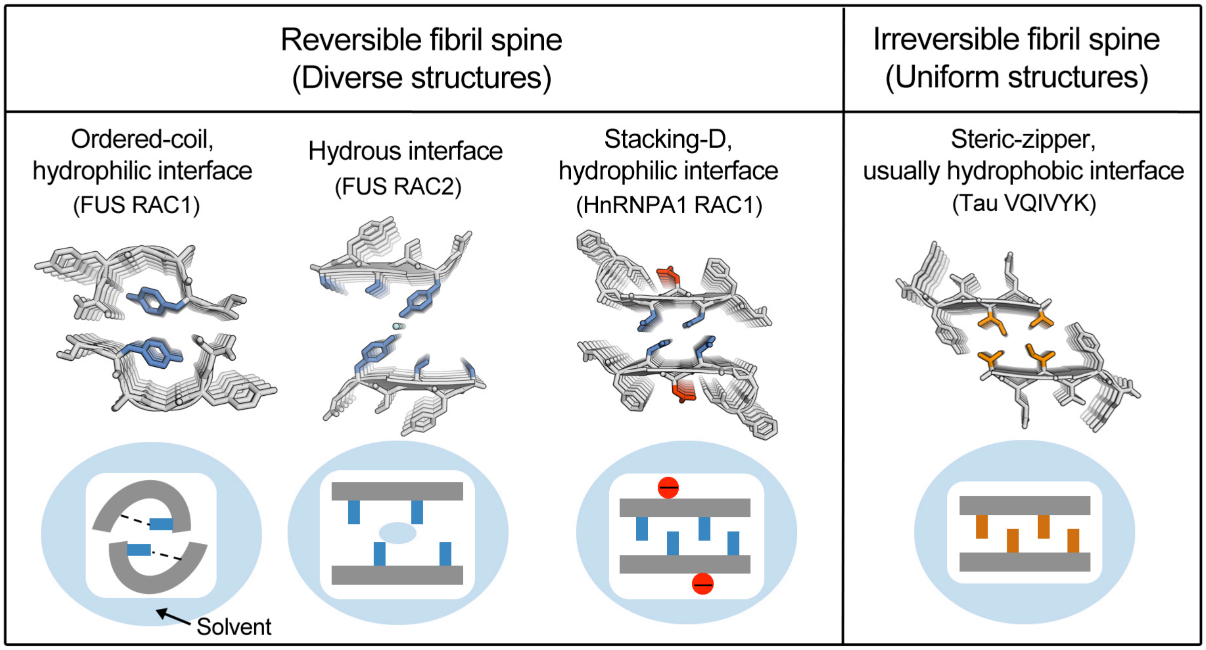

Supplementary Figure 15 Diverse reversible amyloid fibril spine versus uniform irreversible amyloid fibril spine. Representative atomic structures for different structural classes are shown. PDB IDs from left to right: 5XSG, 5XRR, 6J60, 2ON9. Structure cartoons are shown below. Residues involved in hydrophilic interface are in blue; those involved in hydrophobic interface are in orange. Negatively-charged Asp are in red. Peptide back bones are in gray.

Supplementary Table 1 A list of peptides used in this work. The gelation or fibrillation conditions for each peptide are provided.

Name		Sequence	Gelation/fibrillation	
			Concentration (mg ml^{-1})	Condition
		GGFGG	20	4 °C, 48 h
		GGYGG	20	4 °C, 48 h
		GGGFGGN	20	4 °C, 48 h
		GGGYGGS	20	4 °C, 48 h
		RGGFGGS	20	4 °C, 48 h
		NFSGRGGFG	20	4 °C, 48 h
		GGNFSGRGGFGG	20	4 °C, 48 h
		RGGNFSGRG	20	4 °C, 48 h
		GRGGFGGSR	20	4 °C, 48 h
hnRAC1	WT	GFGGNDNFG	3	4 °C, 48 h
	F210A	GAGGNDNFG	20	4 °C, 48 h
	F216A	GFGGNDNAG	20	4 °C, 48 h
	G211V	GFVGNDNFG	0.5	4 °C, 48 h
	D214N	GFGGNNNFG	3	4 °C, 48 h
	D214V	GFGGNVNFG	3	4 °C, 48 h
Ac-hnRAC1*		Ac- GFGGNDNFG	1	4 °C, 48 h
hnRAC2	WT	GFGNDGSNF	15	4 °C, 48 h
	D250V	GFGNVGSNF	5	4 °C, 48 h
	F247A	GAGNDGSNF	20	4 °C, 48 h
Ac-hnRAC2		Ac-GFGNDGSNF	1	4 °C, 48 h
hnRAC3	WT	YNDFGNY	5	4 °C, 24 h
	D262V	YNVFGNY	3	4 °C, 24 h
	D262N	YNNFGNY	3	4 °C, 24 h

*Ac- represents N-terminally acetylated.

Supplementary Note 1: ROSETTA command lines and flags for the modeling of hnRAC2 and hnRAC3.

```
# Command Lines of Rosetta Runn
minirosetta.linuxgccrelease @Flag_File

# The example of Flag_File is as follows:
######################################################################################
-database my_location/minirosetta_database
-run:protocol threading

-in:file:template_pdb < starting pdb coordinate of the monomer backbone >
-symmetry_definition  < location of the symmetry definition file for constructing the fibril >
-in:file:fasta  < fasta file of the amino acid sequence >
-in:file:psipred_ss2 < psipred vformat to specific beta sheet conformation for all the positions >
-loops:frag_sizes 9 3 1
-loops:frag_files < 9-mer and 3-mer fragment files > none

-nstruct 1
-in:file:extended_pose 1
-in:file:fullatom
-loops:remodel quick_ccd
-loops:relax relax
-relax:jump_move true
-loops:constrain_rigid_segments 0.3
-relax:coord_cst_width 2.0
-relax:coord_cst_stdev 1.0
-cm:loop_rebuild_filter 500
######################################################################################
```
